# Cross-talk between AMPK and EGFR dependent Signaling in Non-Small Cell Lung Cancer

**DOI:** 10.1038/srep27514

**Published:** 2016-06-09

**Authors:** Paurush Praveen, Helen Hülsmann, Holger Sültmann, Ruprecht Kuner, Holger Fröhlich

**Affiliations:** 1 University of Bonn, Bonn-Aachen International Center for IT, Dahlmannstr. 2, Bonn Germany; 2 The Microsoft Research-University of Trento Center for Computational and Systems Biology, 38068 Rovereto, Italy; 3 Cancer Genome Research Group, German Cancer Consortium (DKTK), German Center for Lung Research (DZL), German Cancer Research Center (DKFZ), Im Neuenheimer Feld 460,D-69120 Heidelberg, Germany

## Abstract

Lung cancers globally account for 12% of new cancer cases, 85% of these being Non Small Cell Lung Cancer (NSCLC). Therapies like erlotinib target the key player EGFR, which is mutated in about 10% of lung adenocarcinoma. However, drug insensitivity and resistance caused by second mutations in the EGFR or aberrant bypass signaling have evolved as a major challenge in controlling these tumors. Recently, AMPK activation was proposed to sensitize NSCLC cells against erlotinib treatment. However, the underlying mechanism is largely unknown. In this work we aim to unravel the interplay between 20 proteins that were previously associated with EGFR signaling and erlotinib drug sensitivity. The inferred network shows a high level of agreement with protein-protein interactions reported in STRING and HIPPIE databases. It is further experimentally validated with protein measurements. Moreover, predictions derived from our network model fairly agree with somatic mutations and gene expression data from primary lung adenocarcinoma. Altogether our results support the role of AMPK in EGFR signaling and drug sensitivity.

Lung cancer is the second most prevalent cancer in both men and women and accounts for 12% of all new cases of cancers reported worldwide^1^. It caused about 1.5 million deaths globally in 2010^2^ and is the leading cause for cancer deaths^3^. Non small cell lung cancer (NSCLC) is a category of lung cancer in which malignant cells are formed in lung tissues. About 85% of lung cancers are NSCLC, including 40% lung adenocarcinoma (ADC)^4^. Like most types of cancer, lung ADCs are often perceived as a disease resulting from errant inter and intra-cellular communications manipulated by key signaling molecules. Being a highly heterogeneous malignancy, it is important to understand the etiology and pathogenesis of the disease in order to control and treat lung ADCs.

As a critical, disease relevant factor aberrant activation of EGFR dependent signaling has been implicated in lung ADCs^5^,^6^. In consequence several monoclonal antibodies against EGFR have been developed. These include gefitinib (Iressa) and erlotinib (Tarceva). Their efficacy is dependent on L858R/Deletion19 mutation^7^. Many of these therapies induce an initial tumor regression. However, in most cases tumors become insensitive to initial therapies and evolve into more aggressive and resistant phenotypes^8^,^9^. One explanation of the decreased therapeutic benefit is the acquisition of second EGFR mutations, which make cells drug resistant^10^. For example, a T790M mutation occurs in more than 50% of EGFR-mutant lung cancers^11^.

To overcome such treatment failures new targeted therapies need to be developed, possibly within a combinatorial or poly-pharmacological approach^12^,^13^. The reason is that most likely alternative cell signaling molecules are responsible for drug insensitivity and drug resistance. A recent study has shown that activation of AMPK sensitizes EGFR wildtype H1299 cells and xenografts to erlotinib treatment^14^. This synergistic effect was less obvious in EGFR mutated tumor models that may be due to endogenous AMPK activity and solely EGFR TKI (Tyrosine Kinase Inhibitor) sensitivity. This raises the question about possible molecular mechanisms.

In this study we thus focused on the interplay between the AMPK and EGFR dependent signaling cascades in lung ADC. Accordingly, we reconstructed and subsequently validated a network of 20 genes that were associated with erlotinib response or harbor mutations in lung cancer xenograft models^14^. The approach for network reconstruction is based on single siRNA based knockdowns of each gene in the H1650 cell line (EGFR, delE746-A750) and subsequent gene expression profiling. Based on these data we employed Nested Effects Models (NEMs) as a statistical learning approach to unravel key elements of the interplay between AMPK and EGFR dependent signaling^15^. The resulting network is then validated using protein expression data in cell lines and lung ADC patient data (RNAseq plus somatic mutations) from The Cancer Genome Atlas (TCGA), demonstrating the relevance of our findings.

## Results

### Experimental Data

A list of 20 genes was compiled, for which protein expression was associated with EGFR TKI response in patient-derived lung cancer xenografts, or mutations have been observed in these models^14^: In more detail, the proteins coding for mTOR, PRKAA1, RAF1, RPS6KA1, and RPS6KAB1 were differentially expressed in RPPA analysis of NSCLC xenograft models^14^. For the genes BCL10, ESPL1, ITGB4, LEPR, TRUB2, and WDR3 mutations were identified across the same NSCLC xenografts (unpublished data). The remaining genes EGFR, GSK3A, GSK3B, PIK3C3, PRKAB1, SRC, STK11, TSC1, and TSC2 were individually selected from EGFR/ERBB/AMPK signaling pathways (KEGG^16^).

Table 1 shows a characterization of our 20 genes in terms of their Gene Ontology annotation (biological processes), demonstrating an involvement of all proteins into biological processes that are associated with tumor development and cell growth (see full Gene Ontology annotation in file S5).

**Figure 1:**
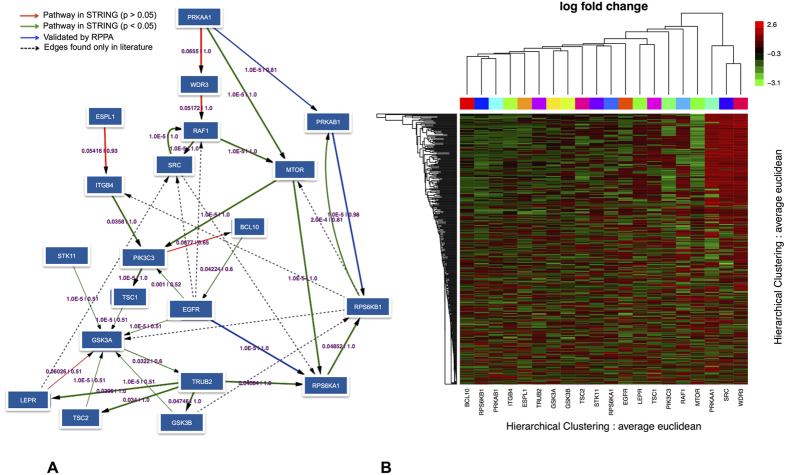
(**A**) High confidence edges (50% probability cutoff) showing the interplay between PRKAA1/PRKAB1 and EGFR dependent signaling. Edges indicate upstream/downstream relations. They may not correspond to direct, physical protein interactions. Edge thickness is drawn according to the level of edge confidence. Edge labels indicate the p-value according to the comparison with the STRING^22^ database (first number) as well as the level of edge stability (from 0 to 1, second number). Green edges indicate a nominell significant (p < 0.05) overlap with pathways from STRING (using only edges with confidence >0.5), while red ones do not. Blue edges are validated by protein expression data. Dashed black edges can only be found as protein-protein interaction in STRING, but are not present in our network. An additional complete validation of our network with protein-protein interactions from the HIPPIE database^23^ can be found in Figure 5 of supplemental file S2. (**B**) The heatmap shows the perturbation effects for individual gene silencings: rows = effects observed, columns = genes perturbed. The colors depicts log fold change for corresponding perturbations (green = down-regulation, red = up-regulation).

**Figure 2:**
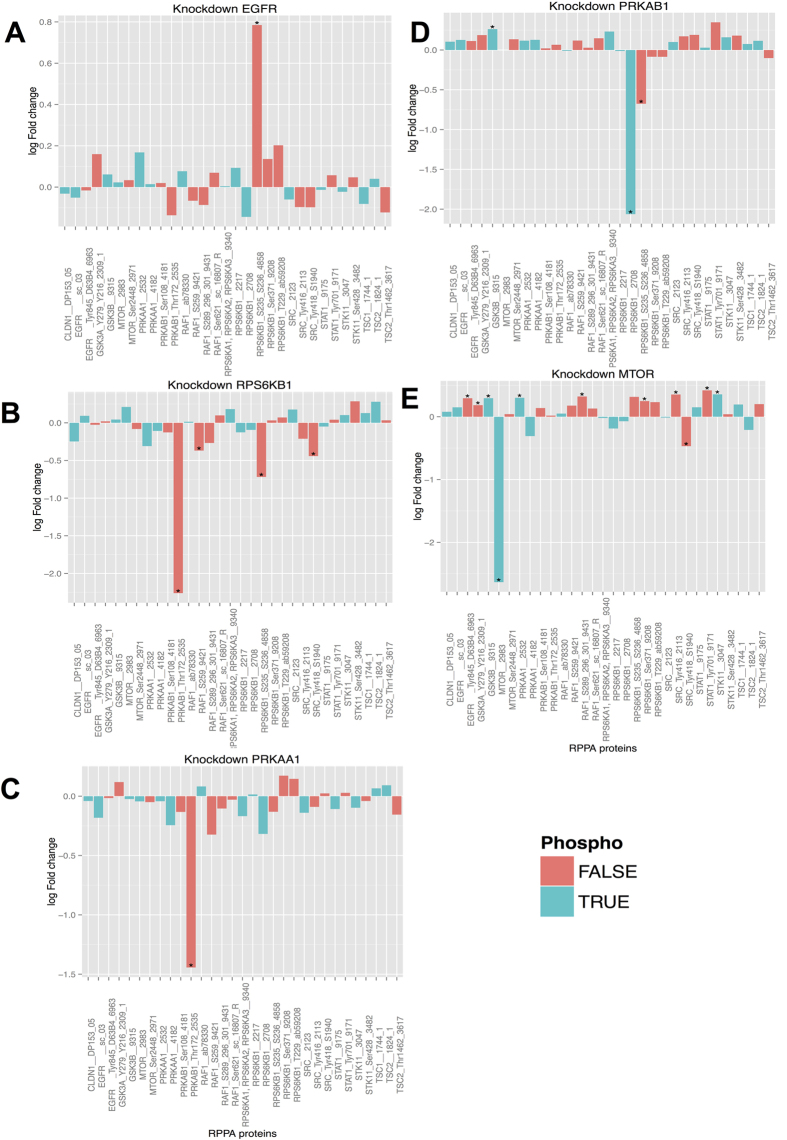
Protein log fold changes after different knockdowns. The RPPA technique is antibody based, and thus for one and the same protein different identifiers can appear on the x-axis. The employed antibody is indicated as part of the protein name. Bar colors indicate total (red) or phospho-protein (blue) concentration changes. A star (*) indicates a statistically significant result (FDR < 5%). Effects are given for knockdown of: EGFR (**A**), RPS6KB (**B**), PRKAA1 (**C**), PRKAB1 (**D**) and mTOR (**E**).

Targeted siRNA-based knockdown experiments were performed in triplicates for each of these 20 genes in H1650 cells (EGFR mutated). The efficiency of each gene knockdown with respect to transcript reduction was verified by qPCR to be >70% compared to non-template controls (Figure 1 in S2 and data in S3 and S4). 72 h after siRNA transfection genome-wide expression profiling was performed. The expression measurements were done in two batches, and all experiments were done in triplicates using the Illumina Whole-Genome Gene Expression Bead Array Chips^17^. The data, exported from the Illumina BeadStudio, is available via the GEO (Gene Expression Omnibus) database (GSE69747). The lumi package^18^ was used to read in and preprocess the data for further analysis, including quantile normalization. To get rid of the batch effect we used the ComBat function from sva package^19^ (Figure 2 in S2).

### Differential Expression Analysis

Limma^20^ was used to analyze expression profiles for each of the 20 gene knockdowns (see details in Materials and Methods). Genes with adjusted p-values < 0.05 and absolute log fold change >1.5 were declared as differentially expressed. For 13 of 20 genes specific microarray probes reflected a transcript reduction of more than 50%. In total 388 genes (see suplementary file S1) from all 20 gene silencing experiments were retrieved. A heatmap of relative expression changes (log fold changes) of these genes is shown in Fig. 1B. For the following our focus was on the reconstruction of the network between the 20 perturbed genes, given the observed transcriptional responses.

### Network Reconstruction

**Figure 3:**
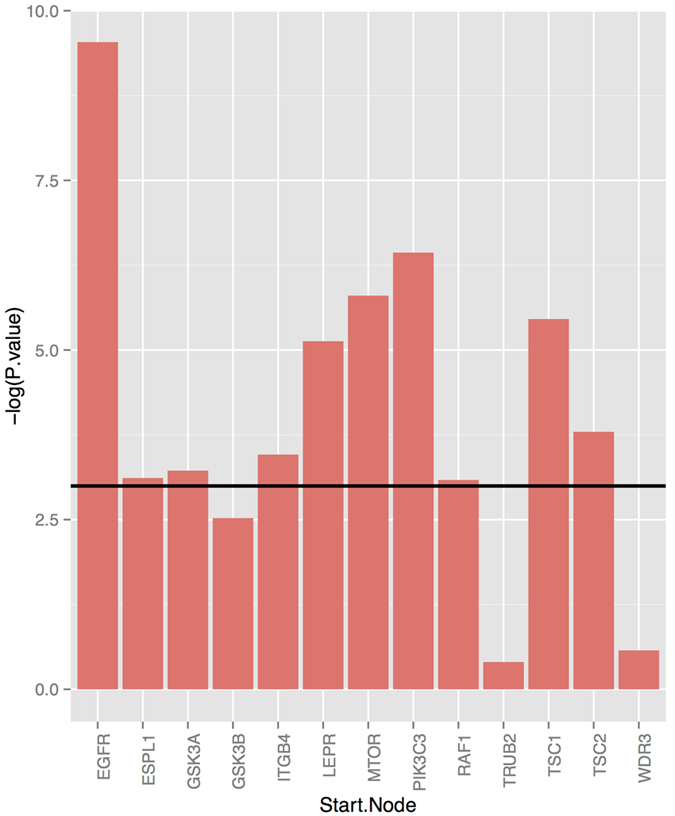
Agreement of inferred network with patient data. The X-axis shows S-genes, for which somatic mutations in patients were observed. The Y-axis depicts the significances (−log p-values) of the overlaps of expected effects on S and E-genes with observed effects (i.e. differentially expressed genes). The horizontal line shows a significance cutoff of 5%.

After the previously described preprocessing and filtering of gene expression data raw p-values were summarized in a 20 × 388 matrix (Figure 3 in S2) and their densities estimated by fitting a mixture model (see Materials and Methods). These data were used to infer a causal network among the 20 silenced genes (henceforth referred as S-(silenced) genes) via Nested Effects Models (NEMs, see Materials and Methods). NEMs are a statistical learning method that aim at finding the most probable, unobserved signaling network given transcriptional response profiles of individual S-gene knockdowns. Edges in a NEM model indicate upstream/downstream relationships. Prior knowledge from several information sources (Gene Ontology, KEGG pathways, protein-protein interactions, protein domains, protein domain interactions) were used and probabilistically integrated at this step via our previously proposed Noisy-OR model^21^.

**Figure 4:**
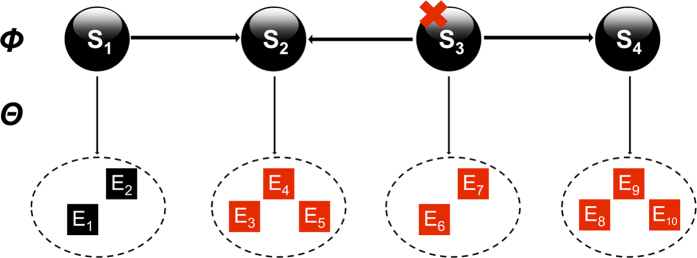
Idea behind Nested Effects Models. Perturbation of S-gene S3 is expected to yield measurable downstream effects on transcriptional reporters E6 and E7. Since S3 is at the same time upstream of S2 and S4, reporters attached to these S-genes are predicted to show an effect. On the other hand, perturbation of S4 does only effect reporters E8, E9, E10. NEM structure learning aims for reconstructing the wiring of the S-genes based on the observable effects on downstream reporters. The attachment of individual reporter genes is treated as an unkown nuisance parameter, which is integrated out, i.e. the network score averages over all possible attachment positions of individual reporters.

The whole NEM network reconstruction was repeated 1000 times while re-sampling from the set of 388 effect reporters (called E-genes in the following) with replacement, yielding a non-parametric bootstrap. The aim of such an approach is to assess the confidence by which individual edges can be learned from data. For the following we focused on edges that could be observed stably with more than 50% probability. The histogram of bootstrap probabilities for individual edges can be found in the supplements (Figure 4 in S2).

As consequence of our analysis we obtained a network (a graph of high confidence edges), which highlighted the possible interplay between EGFR and AMPK dependent signaling [Fig. 1A]. It is worth mentioning that the network reconstruction remained the same, even if different initial filtering criteria to our expression data were applied, indicating the robustness of our network.

### Network is in Agreement with Protein-Protein Interactions Reported in Databases

We checked the agreement of our inferred network with literature reported and high confidence predictions (confidence value larger 0.5) of protein-protein interactions taken from the STRING 10.0 database^22^. For this purpose we then mapped our silenced genes to the genome-wide protein-protein interaction networks and calculated shortest paths between them. This way we could find a mapping of each of our inferred edges to a pathway in the STRING database. Remarkably none of these pathways had more than two edges, i.e. there was at most one intermediate protein (Table 1 in S2).

One may ask, in how far such a result could have been expected by chance. In order to address this question we conducted a statistical permutation test: We randomly shuffled the node labels of the protein-protein interaction network 50,000 times. For each permuted network we then re-mapped our silenced genes and re-calculated shortest path distances. We then counted, in how many cases a shortest path between two mapped silenced genes in the permuted network was shorter than in the original one. This resulted into a p-value for each edge (Fig. 1A). The vast majority of the edges in our inferred network showed a significant p-value, thus demonstrating a significant agreement with known protein-protein interactions.

It is worth mentioning that several of the non-significant edges showed a high boostrap probability (e.g. *WDR*3 → *RAF*1). That means they appear as statistically stable patterns in our data, although the underlying biological mechanism cannot be uniquely identified.

We wondered, in how far the observed agreement of our network with STRING was reproducible when using an alternative database, namely HIPPIE^23^. HIPPIE comprises several other databases of literature reported protein-protein interactions, but does not contain any predictions. Only 14 out of our 20 silenced genes could be mapped to HIPPIE. All but two inferred edges between mapable genes showed a significant agreement with pathways in HIPPIE (Figure 5 in S2). Once again these pathways had all a short length (Table 2 in S2). Altogether our analysis thus demonstrates a good agreement of the inferred network with protein-protein interactions reported in databases.

Figure 1A and Figure 5 in S2, also depict direct protein-protein interactions that were found in STRING and HIPPIE, respectively, but show a bootstrap probability below 50% according to our data driven model. The number of these edges is comparably low, indicating a high coverage of reported protein-protein interactions by our network. It should be noted that a possible (but not necessary) reason for not finding a certain protein-protein interaction with high probability by our model is that indeed that interaction does not take place in our studied cell system.

### Interpretation of the Network

The inferred network presented some interesting relations among genes that have been discussed in literature: PRKAA1 (AMPK Subunit *α*) was found as one of the major upstream regulators of the inferred network. One of the inferred edges is PRKAA1 → WDR3. The WDR gene family has been found to play critical role in cell cycle and apoptosis and gene regulation^24^. WDR3 has been shown to be controlled via P53, and P53 in turn by PRKAA1 in cancer^25^. The inferred edge is thus in agreement with these findings.

PRKAB1 (AMPK Subunit *β*) is a regulatory subunit of the AMP-activated protein kinase, which was inferred to yield identical perturbation effects than PRKAA1 according to our data. These two subunits together influence the ribosomal protein kinase RPS6, which plays an important role in protein synthesis. This is reflected by the path PRKAA1 → PRKAB1 ↔ RPS6KB1 in our network. It has been shown that AMPK can further induce an effect on the RPS6K complex via mTOR in NSCLC^26^,^27^,^28^. Our network contains the path PRKAA1 → mTOR → RPS6KA1 in agreement with these findings.

Subunits of Glycogen synthase kinase-3 (GSK3A and GSK3B) are known for their role in cell division, proliferation, motility and survival together with a number of pathological conditions including cancer^29^. Our network shows GSK3A and GSK3B subunits downstream of EGFR. This is in agreement with the understanding that EGFR stimulation leads to downstream activation of AKT, which in turn influences GSK3^30^,^31^ in normal and cancer cells. The GSK3 complex is activated via phosphorylation by EGFR altering the *Wnt* pathway components in NSCLC^32^. The inferred network maps this in terms of the relationship between the GSK3 complex and EGFR (EGFR → GSK3A ← GSK3B).

EGFR is an upstream activator of PI3K that is frequently altered in cancer^33^. In lung cancers harboring somatic activating mutations in EGFR, PI3-Kinase has been found to be activated by EGFR via a direct binding^34^,^35^. Our network depicted this in terms of a direct edge between EGFR → PIK3C3.

BCL10 is a gene that is known to induce apoptosis and to activate NF-*κ*B^36^. The protein forms a complex with MALT1, which in turn has recently shown to be required for EGFR-induced NF-*κ*B activation and to contribute to EGFR-driven lung cancer progression^37^. RAF1 is a member of the PI3K/AKT signaling cascade, which is known to activate the mTOR pathway^38^. Hence, this is in agreement with our inferred edge RAF1 → mTOR. mTOR itself yields downstream effects on GSK3^39^, which is also consistent with our network.

### Protein Measurements Confirm Inferred Network Features

The response of 107 phospho and total protein concentrations to the knockdown of five selected network genes (EGFR, PRKAA1, PRKAB1, RPS6KB1, mTOR) was measured via Reverse Phase Protein Arrays (RPPA) (Materials and Methods, [Fig. 2]). The data were analyzed using limma. The knockdown for EGFR yielded an up-regulation of the protein kinase RPS6KB1 (Fig. 2A), which is located downstream of EGFR in our network (Fig. 1). The downstream relation EGFR → RPS6KB1 had been inferred with extremely high stability (100%) in our network and our observation at protein level validates this relation. The second knockdown was for protein kinase RPS6KB1 (p70S6K). RPS6KB1 and PRKAB1 form a cycle in our inferred network. Knockdown of RPS6KB1 yielded a ~ four fold down-regulation of PRKAB1 (Fig. 2B). Knockdown of PRKAB1 caused a four fold reduction of RPS6KB1 expression (Fig. 2D). Together this verifies a positive feedback loop between both proteins. Likewise, PRKAA1 knockdown yielded a ~2.8 fold down regulation of PRKAB1 (Fig. 2C), which is in agreement with our inferred edge *PRKAA*1 → *PRKAB*1.

The knockdown of mTOR yielded statistically significant (FDR <5%) expression changes of several network proteins (Fig. 2E). However, only the fold change of mTOR itself (around eight fold) was in a range that would usually be regarded as biologically meaningful. Hence, we abstain from interpreting these data further.

### Network Reconstruction is in Agreement with Patient Data

In order to establish the further relevance of our reconstructed network The Cancer Genome Atlas (TCGA) database was explored to look for patient samples with somatic mutations in lung adenocarcinoma (file S6), resulting in hits for 13 out of the 20 network genes under study. We extracted RNAseq data for these patients as well as for normal samples (*n* = 5). As expected, the number of patients carrying a specific somatic mutation varied rather significantly. We compared gene expression data of patients with a specific somatic mutation to normal samples using voom and limma^40^. This resulted in 97 to ~4300 differentially expressed genes per somatic mutation (FDR <5%). The heatmap of the union of these genes is shown in Figure 9 in S2).

In order to check the agreement of these data with our network we collected for each somatic mutation of one of the network genes all downstream genes, including attached transcriptional effect reporters (see details in Material and Methods). These genes thus comprise the effects predicted by our network. We then tested the overlap with these genes to the genes declared as differentially expressed according to our RNAseq data. The significance of the overlap was assessed using a hyper-geometric test. For 10 out of 13 tested somatic mutations, we could in this way establish a significant overlap with p-value <0.05 [Fig. 3]. This is a remarkable result given the large heterogeneity of patients, the different number of patients carrying a specific somatic mutation and the fact that combinations of somatic mutations may induce in a specific patient rather complex and non-linear changes of gene expression.

## Discussion

Targeted therapy of EGFR is well established for lung ADC, but frequently fails due to acquired drug resistances. Moreover, the existence of alternative signaling pathways could be responsible for an incomplete drug response or complete treatment failure, raising questions about additional treatment options. In that context, AMPK activation has been recently suggested as a possible way to sensitize cells to erlotinib^14^. Our present study aims to shed light on the possible underlying mechanism by focusing on the interplay between EGFR and AMPK dependent signaling cascades. We used siRNA based gene knockdowns together with gene expression profiling to gain insights into the network between 20 proteins, which according to previous experiments showed significant expression changes after AMPK activation and erlotinib treatment. Application of probabilistic network inference using Nested Effects Models yielded a number of high confidence edges, which are largely in agreement with the common literature and further experimental validation data, including protein measurements after siRNA perturbation. Remarkably, our network is also in agreement with patient gene expression and somatic mutation data.

A limitation of our network model is the restriction to 20 selected proteins. While the prior relevance of these proteins has been implicated in previous experiments in model systems^14^, their actual involvement into the human disease pathology is not guaranteed. Also other important proteins might play a role, which are not included into our network. In addition, all experimental data were generated in the context of the cell line H1650, its genome and its activation status of signaling molecules at a specific time point after pertubation. Further studies should focus on the dynamical aspect of signaling cascades by measuring data at several time points.

In summary, our approach allowed us to shed light on the interplay between EGFR and AMPK dependent signaling in a NSCLC model system.

## Materials and Methods

### Cell culture, Treatment and siRNA-based Gene Knockdowns

H1650 cells (2 × 105) were cultured in 2 ml RPMI-1640 medium on 6-well plates and transfected after 24 hours growth using Lipofectamine RNAiMAX Reagent (Life Technologies). Gene-specific siRNAs pools (Dharmacon) and non-template controls (40 nM) were transfected and harvested after 48 h (triplicates). RNA was isolated using the RNeasy Plus 96 Kit according to the manufacturer’s instructions. mRNA gene levels were determined by qPCR using Taqman assays (Life Technologies) and the Lightcycler system (Roche). Knockdown efficiency was determined by delta-delta Ct method using internal control genes (ESD and B2M) and the non-template control as reference sample. siRNAs and qPCR assays are shown in files S3 and S4.

### Reverse Phase Protein Array

H1650 protein lysates upon 72 h gene knockdown (PRKAA1, PRKAB1, EGFR, MTOR and RPS6KB1) were analyzed via Reverse Phase Protein Array as previously described^41^. Quantitative data is given in supplementary data (file S2, Figure 8).

### Limma Analysis

Limma is a linear modeling based technique, which employs an empirical Bayes estimate of the gene wise variance^20^. In this article gene expression was measured in triplicates in a control situation and after siRNA knockdown of a specific gene. Accordingly, a linear model with one factor describing the sample type (control, knockdown of gene 1, knockdown of gene 2, …) was fitted. Afterwards the contrasts to the control group were extracted.

### Estimating Perturbation Effects

P-values reflect the probability to obtain a test statistic at least as extreme as the one observed, given the null hypothesis is true. However, for our network reconstruction we are interested to quantify for each transcript the probability for its perturbance, which is a different quantity. In order to estimate these transcript specific perturbation effects a Beta-Uniform Mixture model (BUM) can be fitted to the observed p-value distributions^42^. The BUM model decomposes the observed p-value distribution into a uniform part (the null distribution) and a second part (the alternative distribution), represented itself by two Beta distributions. Based on the alternative distribution it is now possible to estimate the transcript specific perturbation effect. After applying this technique to each of our 20 gene silencing experiments, we arrived at a 20 × 388 matrix of p-value densities (Figure 3 in S2), which served as the input for the NEM inference explained in the next section.

### Nested Effects Model (NEM)

NEMs (Nested Effects Models) are a class of probabilistic graphical models used to reconstruct a pathway based on measurable downstream response profiles of individual gene knockdowns^15^,^43^. Gene silencing typically alters steady-state expression/activity levels of downstream transcripts^44^,^45^. Accordingly, a NEM has two components: the unknown and not directly observable system of silenced proteins (called S-genes) and the connection of these proteins to downstream effect reporters (E-genes, Fig. 4). NEM network inference is based on a data matrix *D* where *D*_*sk*_ is the effect on E-gene *k* for the knockdown of S-gene *s*. In our case the probabilities for these effects are quantified via p-values densities (see above). Based on the given data for any candidate network structure 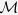 it is possible to calculate a marginal log-likelihood in polynomial time complexity^42^,^43^. This marginal log-likelihood serves as a score for each network structure.

The network score may degrade, if reporter genes are uninformative. We therefore applied a trick suggested by Tresch and Markowetz^46^. More specifically, an isolated, virtual S-gene *null* is added to the network structure. It has been demonstrated that this *null* node filters out irrelevant reporters effectively, because irrelevant reporters have a higher attachment likelihood to *null* than to any other S-gene.

Since the space of hypothetically possible network structures grows at least exponentially with the number of S-genes, heuristic algorithms are required to scan the space of possible candidate structures effectively. In our case we employed the Module Network learning algorithm^42^. The algorithm first splits the overall set of S-genes into smaller groups in a data driven manner. For these groups of S-genes the optimal network structure is found via exhaustive search. In a final step identified sub-networks (modules) are connected using a local search strategy.

The search in network space can be enhanced by incorporating prior knowledge. More specifically, the method proposed earlier by one of the authors^42^ allows to specify for each edge an a priori probability. Here these prior probabilities were estimated from several information sources using the Noisy-OR approach suggested in Praveen and Föhlich^21^. Details about the individual employed information sources can be found in text S2.

The output of the NEM learning procedure consists of a network between S-genes as well as estimated attachments of downstream effect reporters (E-genes). For the data used in this article estimated attachment probabilities are shown as a heatmap in Figure 6 in S2. Figure 7 in S2 gives another view on the data by depicting perturbation effects for each knockdown grouped by their most likely S-gene attachment. The plot specifically shows a larger group of reporters, which are most likely uninformative, i.e. assigned to *null*.

### Predicting Perturbation Effects with NEMs

The NEM models allows for qualitatively predicting the effect of a knockdown of each of the S-genes. According to the NEM model perturbation of a particular S-gene S is supposed to affect.

1. All S-genes reachable from S via directed edges, including S itself.
2. All E-genes attached to these S-genes.

Figure 4 depicts an example of the effects predicted by the silencing of S3 in a toy example with four S-genes.

## Additional Information

**How to cite this article**: Praveen, P. *et al.* Cross-talk between AMPK and EGFR dependent Signaling in Non-Small Cell Lung Cancer. *Sci. Rep.* **6**, 27514; doi: 10.1038/srep27514 (2016).

## Supplementary Material

Supplementary Dataset 1

Supplementary Information

Supplementary Dataset 2

Supplementary Dataset 3

Supplementary Dataset 4

Supplementary Dataset 5

## Figures and Tables

**Table 1:**
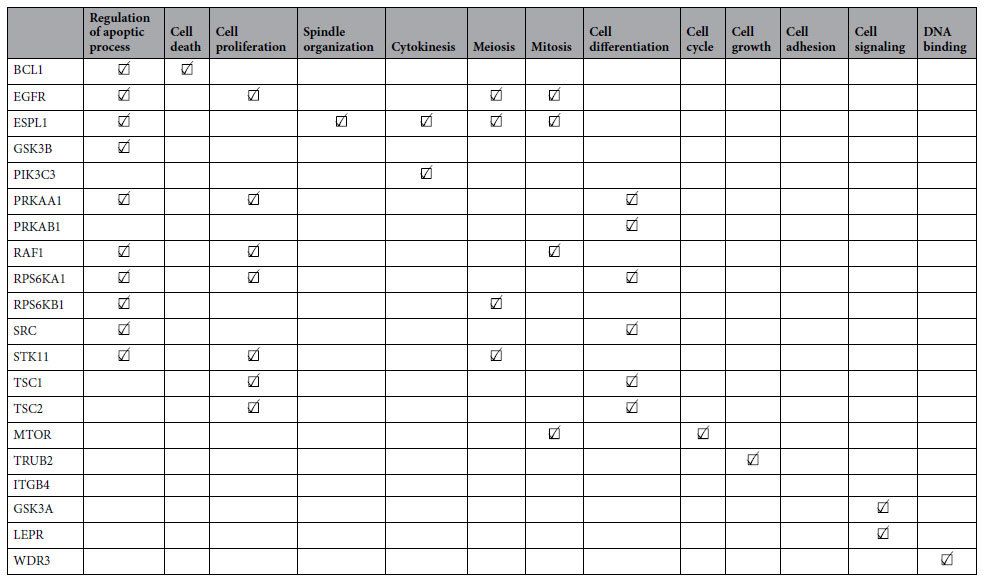
List of genes selected for siRNA based knockdown and annotated GO terms (biological processes).

Marked boxes indicate annotation with respective GO terms (full GO annotation in file S5).
