# Supplementary material for: Cross-talk between AMPK and EGFR dependent Signaling in Non-Small Cell Lung Cancer
Source: Sci Rep. 2016 Jun 9;6:27514. doi: 10.1038/srep27514 (PMC4899720; doi:10.1038/srep27514)
Supplement: Supplementary Information [file srep27514-s2.pdf]

# Cross-talk between AMPK and EGFR dependent Signaling in Non-Small Cell Lung Cancer (Supplement)

Paurush Praveen, Helen Hülsmann, Holger Sültman,
Ruprecht Kuner and Holger Fröhlich

July 14, 2015

## 1 Sources of prior knowledge

The approaches presented here primary consider the following sources of biological knowledge.

### Protein-Protein Interactions database

Protein-protein interaction (PPI) data present the current knowledge about pairs of proteins that interact in living system and hence can be an important source of information as a network prior. Such knowledge resides in various databases, like IntAct, HPRD *etc*. Here we use interaction data from the PathwaysCommons database; a collection of publicly available pathway information [1]. To compute a confidence value for each interaction between a pair of genes/proteins we look at the shortest path distance between the two entities. To calculate the shortest path distance between two nodes the function $sp.between$ function based on Dijkstra's algorithm is used from R-package RBGL. The edge confidence is then computed as the inverse shortest path distance.

### KEGG pathway

KEGG pathways [5] is a knowledge base representing our knowledge on the molecular interaction and reaction networks. It includes various kinds of pathways e.g. metabolic pathways, disease related pathways etc. We compiled a comprehensive network from KEGG with $\sim$3776 nodes and $\sim$29878 edges by merging $\sim$80 KEGG graphs. The Dijkastra algorithm was executed on this graph to compute scores similar to those obtained for PPI databases.

### Gene Ontology

The Gene Ontology (GO) offers controlled vocabularies for aiding the annotation of biomolecules. Interacting proteins often function in the same biological process. This implies that two proteins acting in the same biological process are more likely to interact than two proteins involved in different processes. Here we use this information based on GO *BiologicalProcess* (BP) annotations.Therefore, exploring the knowledge buried in GO annotations seems a promising approach to map relations among genes. To do this mapping of relations comparison of individual GO terms was performed via Lin's similarity measure [6] via the default method in GOSim [3, 10].

### Protein Domain Annotation

It has been found that found that proteins in distinct KEGG pathways are enriched for certain protein domains, i.e. proteins with similar domains are more likely to act in similar biological pathways [4, 2]. Therefore, the confidence for interaction between two proteins can thus be seen as a function of the similarity of the inter-pro domain annotations [7] of proteins. For each protein we constructed a binary vector, where each component represents one Inter-Pro domain. A "1" in a component thus indicates that the protein is annotated with the corresponding domain. Otherwise a "0" is filled in. The similarity between two binary vectors $u$, $v$ (domain signatures) is presented in terms of the cosine similarity

$$S_{domain} = \frac{\langle u, v \rangle}{\|u\| \|v\|} \tag{1}$$

### Domain-Domain Interactions

Two proteins are more likely to interact if they contain domains, which can potentially interact. The DOMINE database collates known and predicted domain–domain interactions [9]. Calculation for edge confidence ($I_{AB}$) based on the DOMINE database is done as

$$I_{AB} = \frac{H}{D_A . D_B} \tag{2}$$

where H is the number of hit pairs found in the DOMINE database and $D_A$ and $D_B$ are the the number of domains in proteins A and B, respectively.

## 2 Methods to compute priors

The Noisy-OR model (NOM) [8] to compute consensus probabilistic prior from the sources described above. Here we describe the NOM approach: The Noisy-OR represents a non-deterministic disjunctive relation between an effect and its

possible causes and has been extensively used in artificial intelligence. The Noisy-OR model assumes that the relation among the causes and the effect is non-deterministic, allowing the presence of the effect in absence of any of the modeled causes. The Noisy-OR principle is governed by two hallmarks: First, each cause has a probability to produce the effect and second, the probability of each cause being sufficient to produce the effect is independent of the presence of other causes .

In our case $X_{ij}^{(1)}$, $X_{ij}^{(2)}$, ..., $X_{ij}^{(n)}$ are interpreted as causes and $\hat{\Phi}_{ij}$ as effect. The link between both is given by

$$\hat{\Phi}_{ij} = 1 - \prod_{k}(1 - X_{ij}^{(k)}) \tag{3}$$

In consequence $\hat{\Phi}_{ij}$ becomes close to 1, if the edge $i \to j$ has a high confidence in at least one knowledge source, because then the product gets close to 0. Hence, in the Noisy-OR model high edge confidences in one information source can overrule low confidences in other information sources. For a detailed description please see Praveen et al. 2013 [8]
This prior was percieved as the S-gene prior for the NEM algorithm together with the data.

# References


[1] Ethan G Cerami, Benjamin E Gross, Emek Demir, Igor Rodchenkov, Ãzagin Babur, Nadia Anwar, Nikolaus Schultz, Gary D Bader, and Chris Sander. Pathway commons, a web resource for biological pathway data. *Nucleic Acids Research*, 39:D685–D690, 2011.

[2] Holger Fröhlich, Mark Fellman, Holger Sültman, and Tim Beißbarth. Predicting pathway membership via domain sinatures. *Bioinformatics*, 24:2137–2142, 2008.

[3] Holger Fröhlich, Mark Fellman, Holger Sültman, Annemarie Poustka, and Tim Beißbarth. Large scale statistical inference of singnaling pathways from rnai and microarray data. *BMC Bioinformatics*, 8(386), October 2007.

[4] Florian Hahne, Alexander Mehrle, Dorit Arlt, Annemarie Poustka, Stefan Wiemann, and Tim Beißbarth. Extending pathways based on gene lists using interpro domain signatures. *BMC Bioinformatics*, 9(1):3, 2008.

[5] Minoru Kanehisa, Susumu Goto, Yoko Sato, Miho Furumichi, and Mao Tanabe. Kegg for integration and interpretation of large-scale molecular data sets. *Nucleic Acids Research*, 2011.

[6] Dekang Lin. An information-theoretic definition of similarity. In *In Proceedings of the 15th International Conference on Machine Learning*, pages 296–304. Morgan Kaufmann, 1998.

[7] Nicola J Mulder, Rolf Apweiler, Terri K Attwood, Amos Bairoch, Alex Bateman, David Binns, Margaret Biswas, Paul Bradley, Peer Bork, Phillip Bucher, Richard Copley, Emmanuel Courcelle, Richard Durbin, Laurent Falquet, Wolfgang Fleischmann, Jerome Gouzy, Sam Griffith-Jones, Daniel Haft, Henning Hermjakob, Nicolas Hulo, Daniel Kahn, Alexander Kanapin, Maria Krestyaninova, Rodrigo Lopez, Ivica Letunic, Sandra Orchard, Marco Pagni, David Peyruc, Chris P Ponting, Florence Servant, Christian J A Sigrist, and InterPro Consortium. Interpro: an integrated documentation resource for protein families, domains and functional sites. *Brief Bioinform*, 3(3):225–235, Sep 2002.

[8] Paurush Praveen and Holger Fröhlich. Boosting probabilistic graphical model inference by incorporating prior knowledge from multiple sources. *PLoS ONE*, 8(6):e67410, 06 2013.

[9] B. Raghavachari, A. Tasneem, T. M. Przytycka, and R. Jothi. Domine: a database of protein domain interactions. *Nucleic Acids Res*, 36(Database issue):D656–61, 2008.

[10] Andreas Schlicker, Francisco S Domingues, Jörg Rahnenführer, and Thomas Lengauer. A new measure for functional similarity of gene products based on gene ontology. *BMC Bioinformatics*, 7:302, 2006.

# Cross-talk between AMPK and EGFR dependent Signaling in Non-Small Cell Lung Cancer (Supplementary Figures)

Paurush Praveen, Helen Hülsmann, Holger Sültman,
Ruprecht Kuner and Holger Fröhlich

March 11, 2016

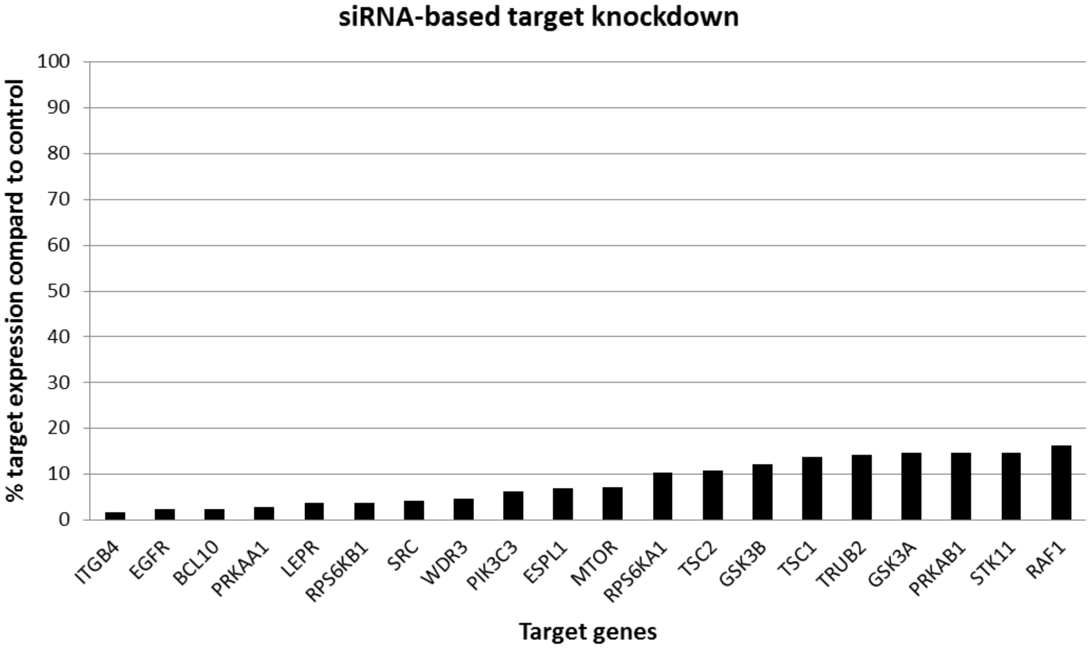


SF1: Target gene expression upon siRNA-based knockdown experiment in H1650 cells. Gene expression was measured by qPCR. Relative expression decrease upon knockdown was calculated in comparison to non-template controls (100%).

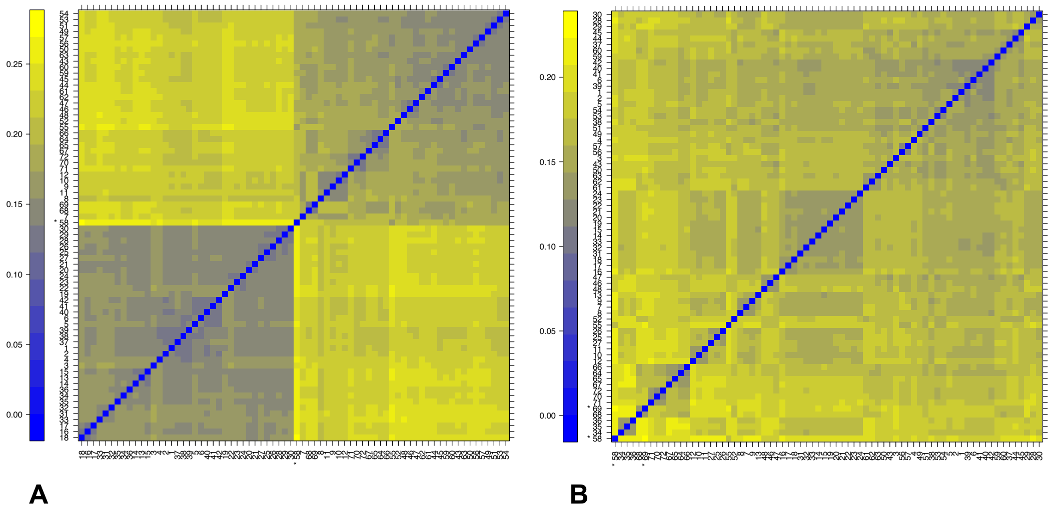


SF2: Batch effect removal from data (A) Heat for the mRNA data before removing the batch effect (B) After removing the batch effect

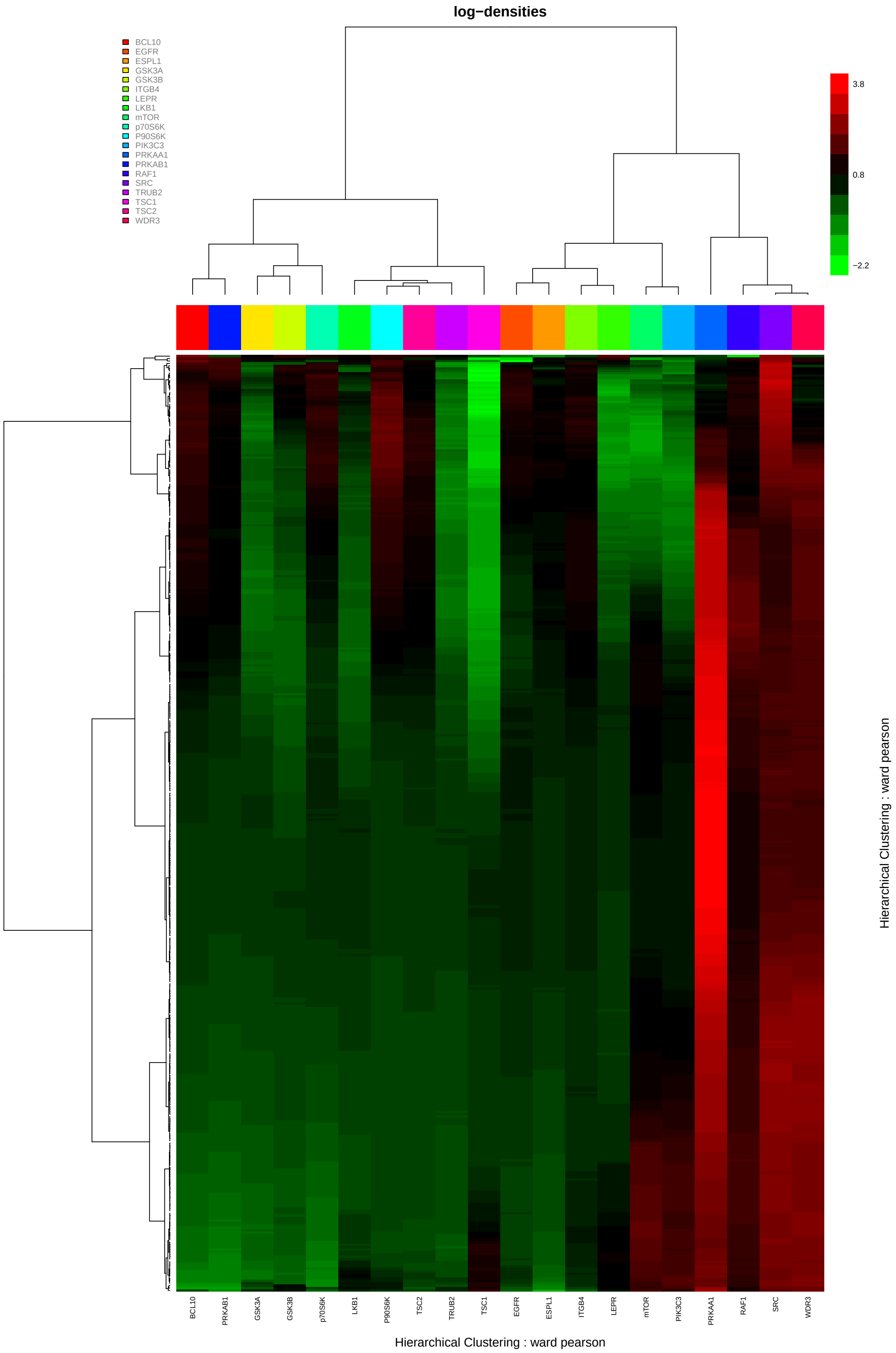


SF3: Heatmap for the log p-value density in mRNA perturbation data

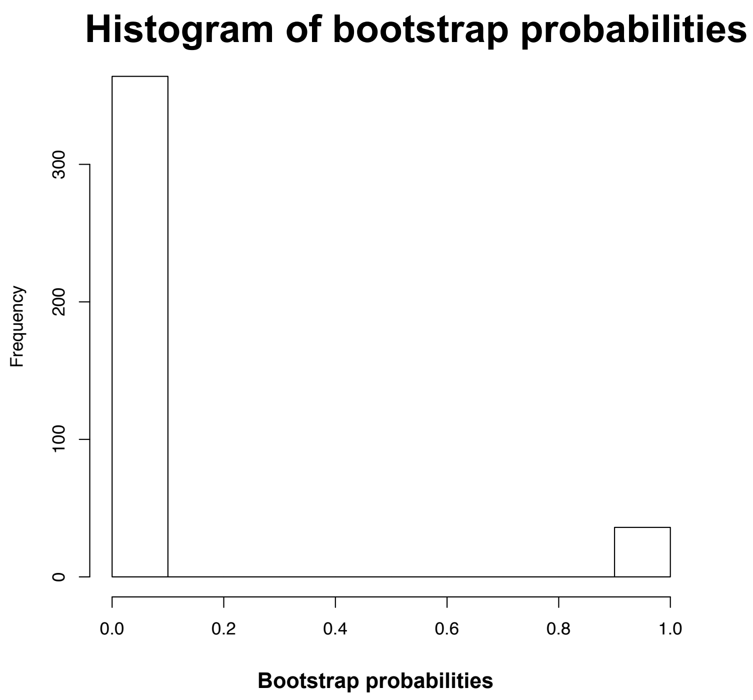


SF4: Histogram for the bootstrap confidence in the inferred bootstrapped network

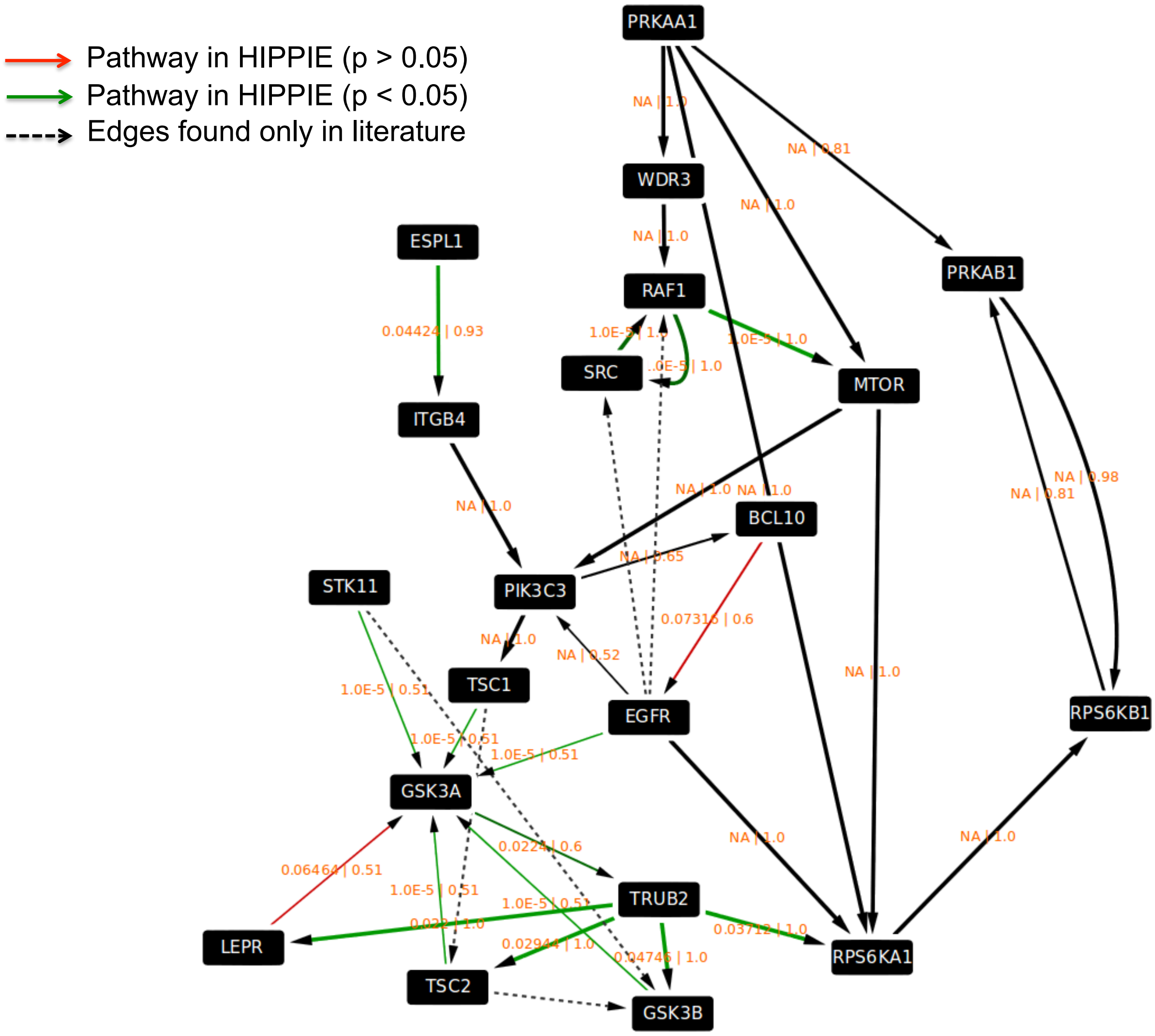


SF5: Comparison of inferred network with HIPPIE

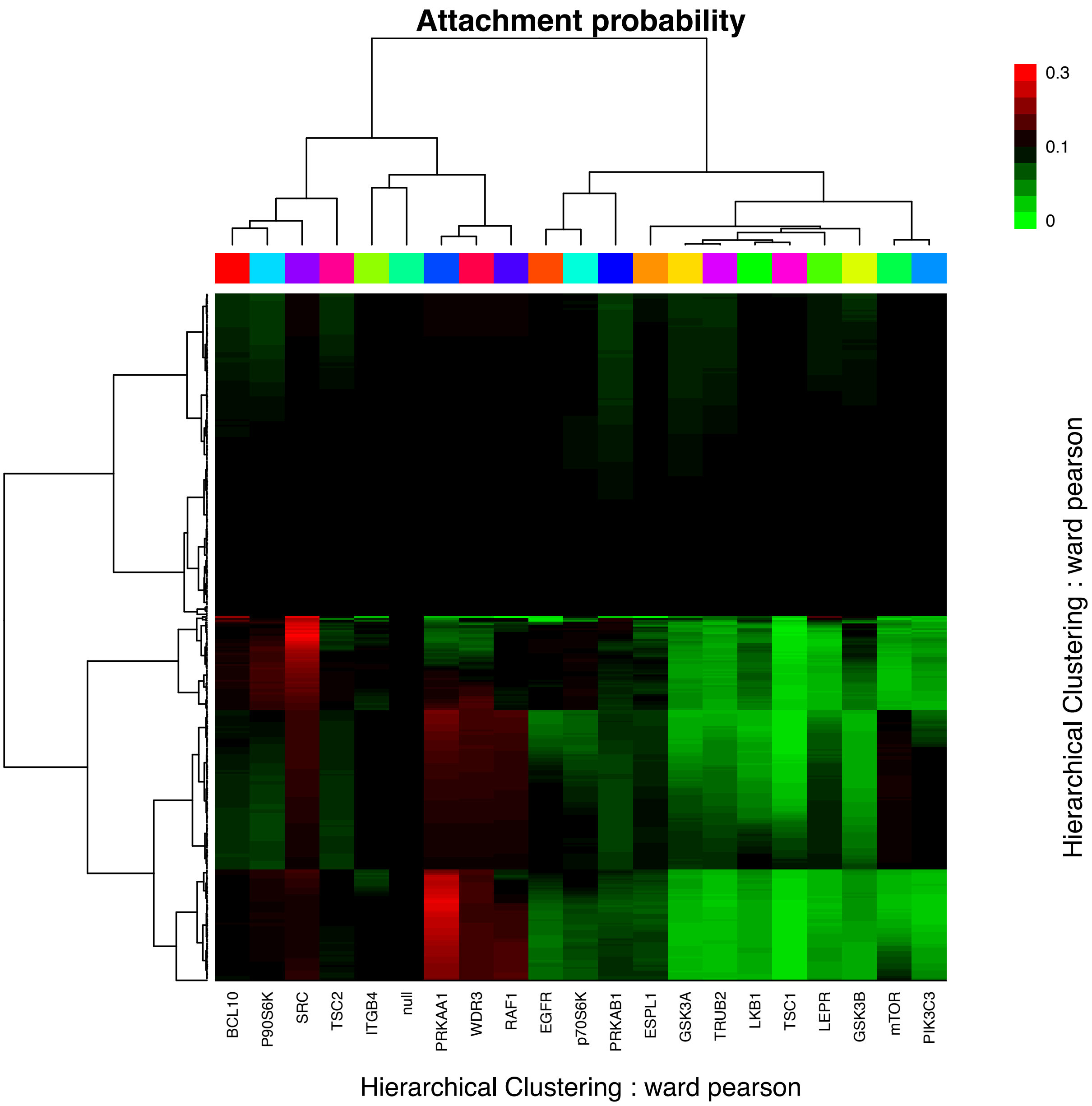


SF6: Heatmap of attachment probabilities of reporter genes

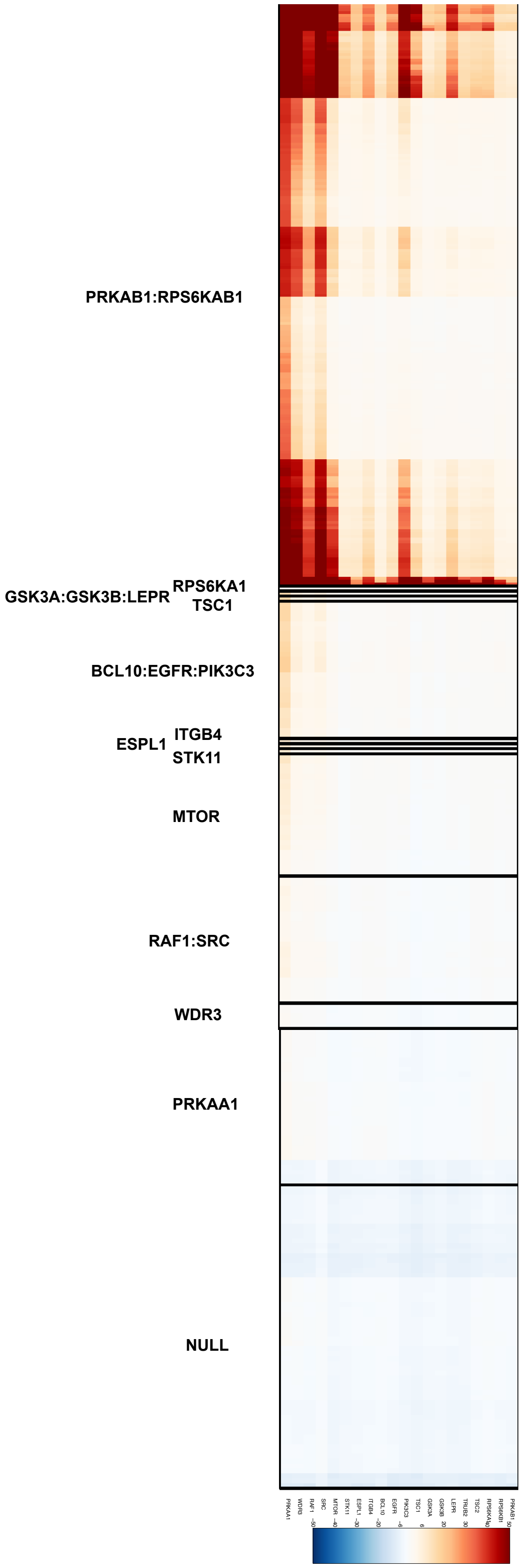


SF7: Heatmap of perturbation effects grouped by mostlikely S-gene attachments.

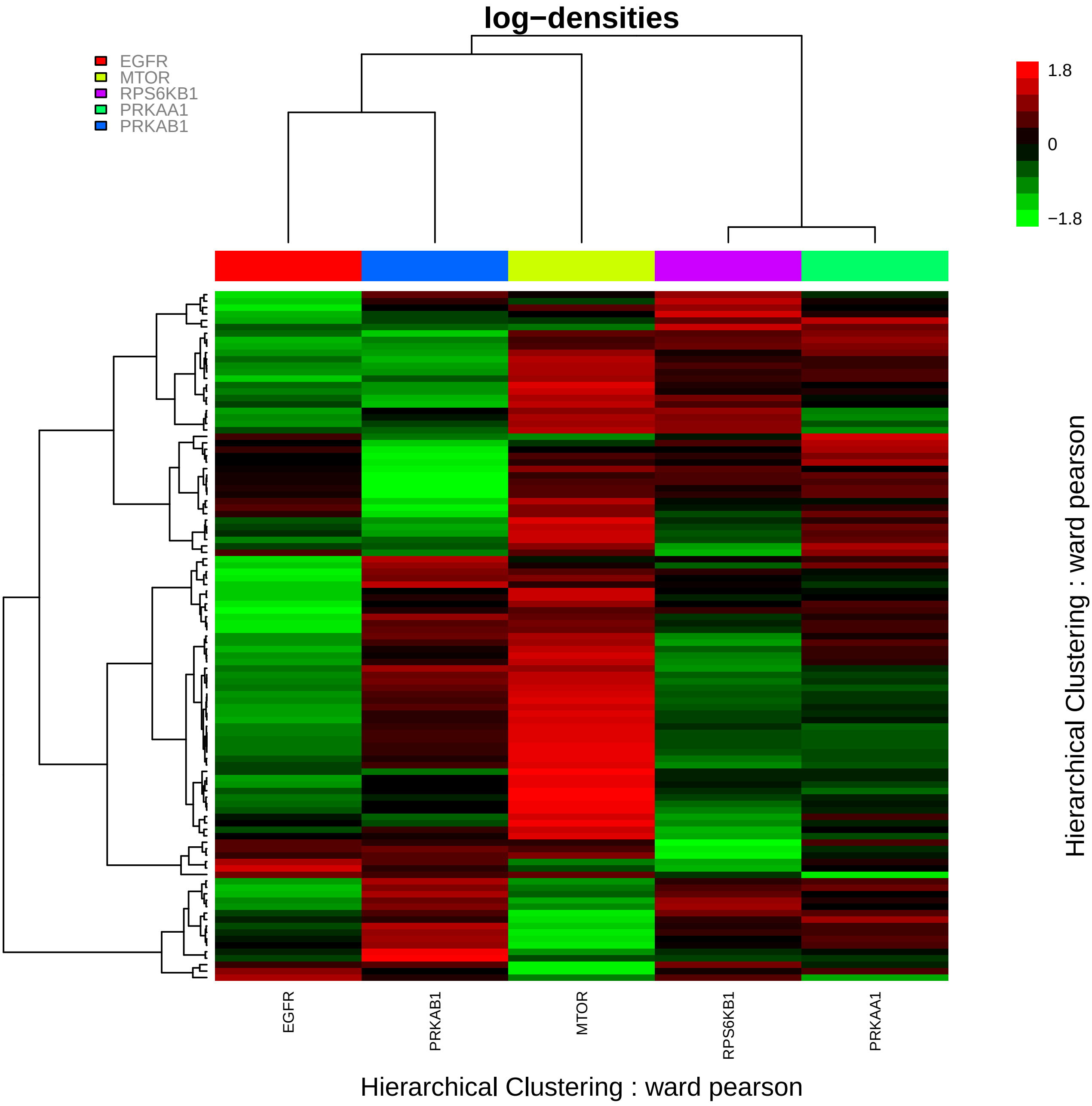


SF8: Heatmap showing effect on proteins for knocking down 5 genes

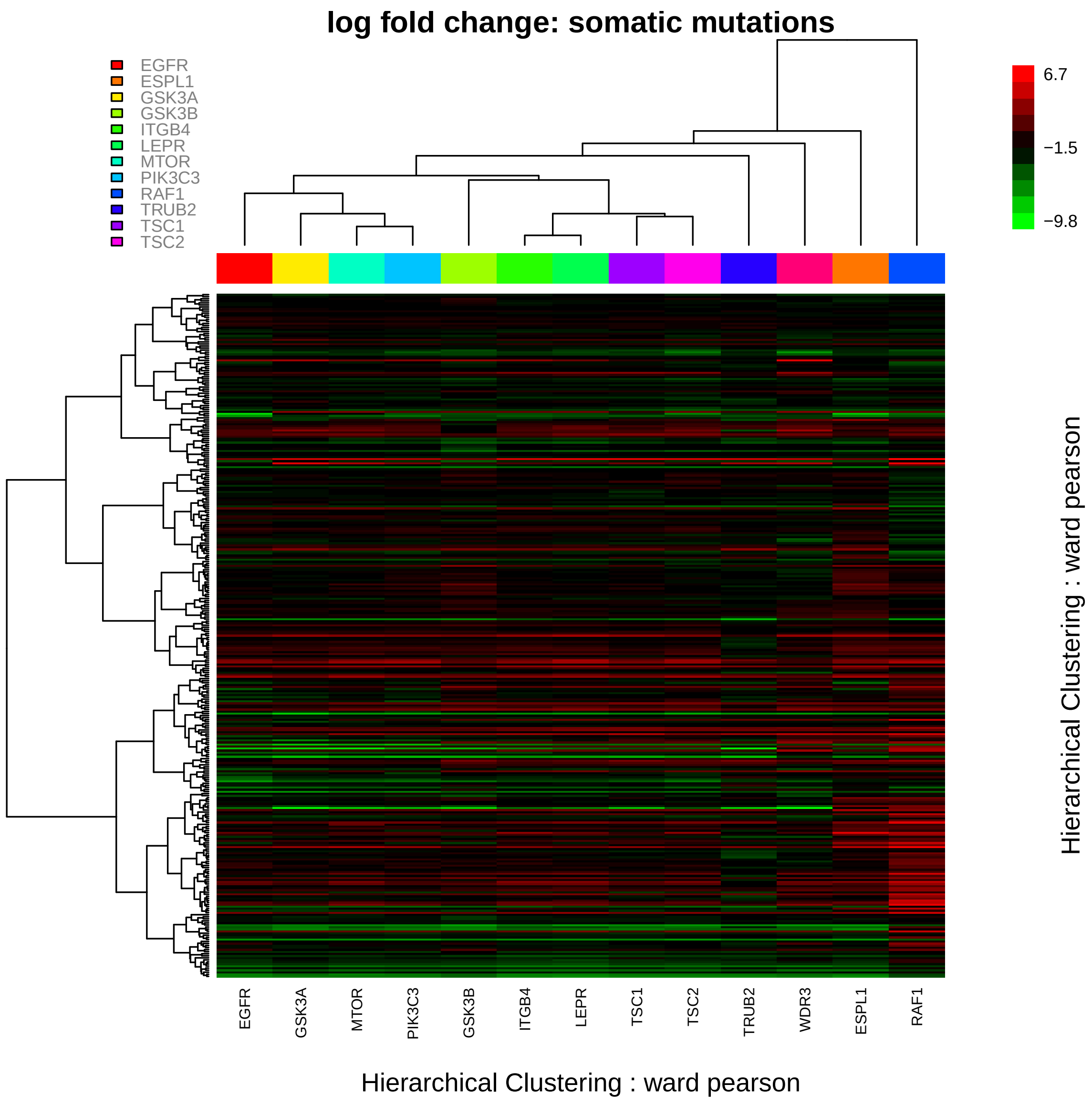


SF9: Heatmap of log fold changes for genes showing differential expression between patients with and without the somatic mutation shown in columns.

Table 1: Inferred network edges explained by STRING paths

| Network Path | Explained by (String) |
|---|---|
| PRKAA1→ PRKAB1 | PRKAA1→PRKAB1 |
| PRKAA1→MTOR | PRKAA1→MTOR |
| PRKAA1→RPS6KA1 | PRKAA1→RPS6KA1 |
| PRKAB1→WDR3 | PRKAB1→RPS6→WDR3 |
| PRKAB1→RPS6KB1 | PRKAB1→RPS6KB1 |
| ESPL1→ITGB4 | ESPL1→CDKN2A→ITGB4 |
| WDR3→RAF1 | WDR3→GNB2L1→RAF1 |
| RAF1→SRC | RAF1→SRC |
| RAF1→MTOR | RAF1→MTOR |
| ITGB4→PIK3C3 | ITGB4→CDKN2A→PIK3C3 |
| SRC→RAF1 | SRC→RAF1 |
| MTOR→PIK3C3 | MTOR→PIK3C3 |
| MTOR→RPS6KA1 | MTOR→RPS6KA1 |
| PIK3C3→TSC1 | PIK3C3→TSC1 |
| TSC1→GSK3A | TSC1→GSK3A |
| TSC2→GSK3A | TSC2→GSK3A |
| BCL10→EGFR | BCL10→CDKN2A→EGFR |
| BCL10→RPS6KA1 | BCL10→RPS6KA1 |
| EGFR→PIK3C3 | EGFR→PIK3C3 |
| EGFR→GSK3A | EGFR→GSK3A |
| STK11→GSK3A | STK11→GSK3A |
| GSK3A→TRUB2 | GSK3A→PRKAG2→TRUB2 |
| TRUB2→TSC2 | TRUB2→PRKAG2→TSC2 |
| TRUB2→GSK3B | TRUB2→PRKAG2→GSK3B |
| TRUB2→LEPR | TRUB2→PRKAG2→LEPR |
| TRUB2→RPS6KA1 | TRUB2→UBC→RPS6KA1 |
| LEPR→GSK3A | LEPR→STAT1→GSK3A |
| RPS6KA1→RPS6KB1 | RPS6KA1→CREB1→RPS6KB1 |

Table 2: Inferred network edges explained by HIPPIE paths

| Network Path | Explained by (Hippie) |
|---|---|
| PRKAA1→ PRKAB1 | NA |
| PRKAA1→MTOR | NA |
| PRKAA1→RPS6KA1 | NA |
| PRKAB1→WDR3 | NA |
| PRKAB1→RPS6KB1 | NA |
| ESPL1→ITGB4 | NA |
| WDR3→RAF1 | NA |
| RAF1→SRC | RAF1→ARRB2→SRC |
| RAF1→MTOR | RAF1→HSP74→MTOR |
| ITGB4→PIK3C3 | NA |
| SRC→RAF1 | SRC→RAF1 |
| MTOR→PIK3C3 | NA |
| MTOR→RPS6KA1 | NA |
| PIK3C3→TSC1 | NA |
| TSC1→GSK3A | TSC1→AKT1→GSK3A |
| TSC2→GSK3A | TSC2→RRAGB→A4→GSK3A |
| BCL10→EGFR | BCL10→UB2V2→EGFR |
| BCL10→RPS6KA1 | NA |
| EGFR→PIK3C3 | NA |
| EGFR→GSK3A | EGFR→AKT1→GSK3A |
| STK11→GSK3A | STK11→A4→GSK3A |
| GSK3A→TRUB2 | GSK3A→EBP2→H11→TRUB2 |
| TRUB2→TSC2 | TRUB2→FYCO1→KINH→1433G→TSC2 |
| TRUB2→GSK3B | TRUB2→FYCO1→LMNA→TOP2A→GSK3B |
| TRUB2→LEPR | TRUB2→FYCO1→RFA1→XRN1→ LEPR |
| TRUB2→RPS6KA1 | NA |
| LEPR→GSK3A | LEPR→GRB2→FCG2B→GSK3A |
| RPS6KA1→RPS6KB1 | NA |
